# Site-specific covalent modifications of human insulin by catechol estrogens: Reactivity and induced structural and functional changes

**DOI:** 10.1038/srep28804

**Published:** 2016-06-29

**Authors:** Ming-Chun Ku, Chieh-Ming Fang, Juei-Tang Cheng, Huei-Chen Liang, Tzu-Fan Wang, Chih-Hsing Wu, Chiao-Chen Chen, Jung-Hsiang Tai, Shu-Hui Chen

**Affiliations:** 1 Department of Chemistry, National Cheng Kung University, Tainan, Taiwan, ROC; 2 Department of Medical Research, Chi-Mei Medical Center, Yong-Kang, Tainan, Taiwan, ROC; 3 Department of Family Medicine, College of Medicine, National Cheng Kung University Hospital, Tainan, Taiwan, ROC; 4 Division of Infectious Diseases and Immunology, Institute of Biomedical Sciences, Academia Sinica, Taipei, Taiwan.

## Abstract

Proteins, covalently modified by catechol estrogens (CEs), were identified recently from the blood serum of diabetic patients and referred to as estrogenized proteins. Estrogenization of circulating insulin may occur and affect its molecular functioning. Here, the chemical reactivity of CEs towards specific amino acid residues of proteins and the structural and functional changes induced by the estrogenization of insulin were studied using cyclic voltammetry, liquid chromatography-mass spectrometry, circular dichroism spectroscopy, molecular modeling, and bioassays. Our results indicate that CEs, namely, 2- and 4-hydroxyl estrogens, were thermodynamically and kinetically more reactive than the catechol moiety. Upon co-incubation, intact insulin formed a substantial number of adducts with one or multiple CEs via covalent conjugation at its Cys 7 in the A or B chain, as well as at His10 or Lys29 in the B chain. Such conjugation was coupled with the cleavage of inter-chain disulfide linkages. Estrogenization on these sites may block the receptor-binding pockets of insulin. Insulin signaling and glucose uptake levels were lower in MCF-7 cells treated with modified insulin than in cells treated with native insulin. Taken together, our findings demonstrate that insulin molecules are susceptible to active estrogenization, and that such modification may alter the action of insulin.

Various clinical observations and experimental data suggest that the interaction between insulin and estrogens affects carbohydrate metabolism^1^. At physiological concentrations, estradiol enhances glucose uptake in adipocytes. However, higher or lower estradiol concentrations may adversely affect the action of insulin^2^. A dual modulatory effect of estrogens on the release of insulin has been proposed. This would involve direct enhancement by an interaction with the cytosolic estrogen receptor, and indirect inhibition upon hydroxylation of estrogens to catechol estrogens (CEs), presumably *via* interaction with alpha-2 adrenergic receptors^3^. However, unlike estrogens that have specific receptors, CEs are not known to have specific receptors.

CEs generated by estrogen metabolism are thought to be endogenous genotoxic agents that target macromolecules. CEs are converted to secretable methoxy derivatives *via O*-methylation by catechol-*O*-methyltransferase^4^. Nevertheless, significant amounts of CE species can be detected by liquid chromatography-mass spectrometry (LC-MS) in human blood^5^ and urine^6^, and in cultured cells^7^. Furthermore, in the blood of diabetic patients who have insulin resistance syndrome, we have recently identified serum proteins that are covalently modified by CEs^8^. An abnormal level of CEs is likely to be present in patients with disturbed homeostasis. However, it is unknown whether CEs form covalent bonds with insulin *in vivo*, for example, when insulin is released endogenously or taken as a medicine, and whether such modification could induce functional changes in insulin molecules, altering normal cell physiology.

When CEs are oxidized to highly reactive 2- and 4-estrogen quinone species (CE-Q) by enzymes or reactive oxygen species, CE-Qs can form adducts with nucleophilic DNA bases^9^ *via* Michael addition and can initiate estrogen-induced tumorigenesis^10^. Modification of proteins by CE-Qs, referred to here as ‘estrogenization’^8^, is less understood than such modification of DNA. During early investigations, either direct or displacement radioisotope labeling^11^,^12^,^13^,^14^ was used to detect the binding of CE-Qs to tubulin, or to some microsomal proteins. Such binding was proposed to impair mitotic spindle formation, and contribute to chromosomal nondisjunction and the induction of aneuploidy. However, these assays suffered from low sensitivity and precision, and were insufficiently powerful to reveal specific sites on proteins modified by CEs. Using modern LC-MS techniques, it was demonstrated that CEs can form covalent bonds with cysteine residues in neuroglobin, which was used as a model protein^15^. Using shotgun proteomics without affinity enrichment, CE adducts were identified on highly abundant serum proteins, such as human serum albumin and immunoglobulins, in the blood sera of diabetic patients who had insulin resistance syndrome^8^. Identification of post-translational modifications using LC-MS-based proteomics remains a challenge for low abundance proteins. However, depending on protein structure, site-specific estrogenization may occur in less abundant proteins provided that their vulnerable residues are accessible to CEs and that the microenvironments of the modification sites favor the reaction.

In this study, we characterized the reactivity of CEs, namely 2- and 4-hydroxyl estrogens (2OHE2 and 4OHE2), towards certain amino acids (AAs) and therapeutic insulin (Humulin R) under normal physiological conditions. Humulin R is a recombinant protein with the same AA sequence as endogenous human insulin. Because disulfide linkages are potential targets for CEs, native digestion combined with the LC-MS^2^ technique was utilized. Multiple fragmentation protocols were applied for in-depth structural characterization, including the commonly used collision-induced dissociation (CID) and advanced electron transfer dissociation (ETD)^16^ techniques that are able to cleave the disulfide linkage and preserve the modified moiety in the gas phase. Estrogenization-induced changes in the structure of insulin were simulated by molecular modeling. The effects of estrogenization on glucose uptake and cell signaling were studied using cultured MCF-7 cells. The purpose of the present work was to provide new data related to the potential impact of insulin estrogenization.

## Results

### Reactivity of CEs with amino acid residues

**Figure 1:**
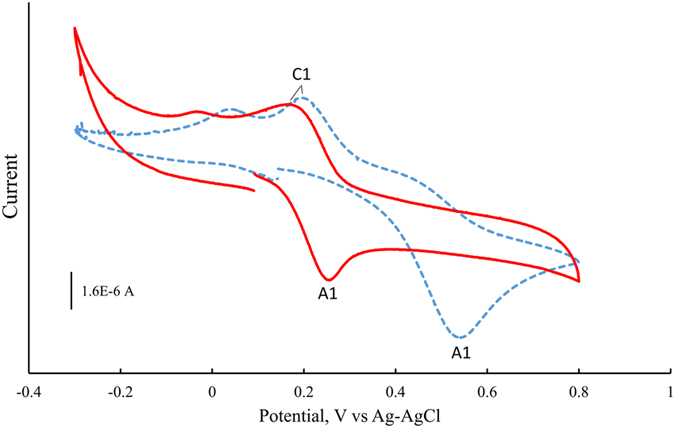
Cyclic voltammetry of catechol (dashed line) versus 4OHE2 (solid line).

To determine whether CEs are easy to activate under ambient conditions, cyclic voltammetry graphs were examined for catechol, redox moieties of CEs, and 4OHE2. Figure 1 displays a two-electron oxidation wave (A1) for catechol at 0.5 V *vs.* Ag/AgCl. This corresponds with the transformation of catechol to *o*-benzoquinone, as reported previously^17^,^18^, and one reduction wave at 0.19 V (C1) *vs*. Ag/AgCl. A shoulder peak near C1 was observed and it may be attributed to minor constitutes of de-protonated catechols (QH^−^ or Q^2−^) present in the un-buffered solution^18^. For 4OHE2, the two-electron oxidative wave (A1) corresponding to the transformation of CE to CE-Q decreases by 0.25 V (from 0.5 V to 0.25 V), resulting in a huge decrease in the peak-to-peak separation (from 0.31 V to 0.07 V) between A1 and C1 as compared to catechol, indicating a higher reactivity or a lower activation energy than that of the catechol moiety.

**Figure 2:**
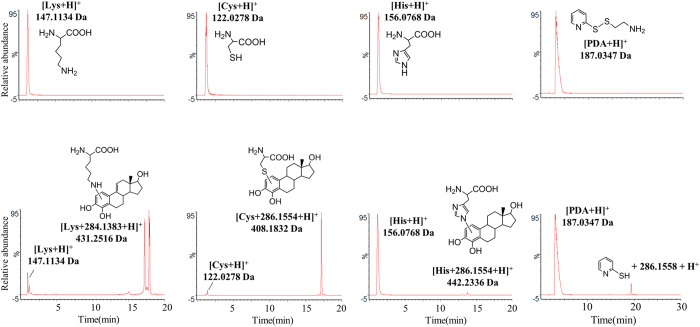
Covalent conjugation with 4OHE2 via co-incubation in PBS buffer. XICs of 4OHE2-conjugated lysine, cysteine, histidine, and PDA (from the left to the right) before co-incubation (top) and after co-incubation (bottom). Modified AAs were all detected after co-incubation.

To study the reactivity of CEs with AAs under normal physiological conditions, a panel of AAs, including those with electron donating (Cys, Gln, Lys, Arg, His, Glu, and Trp) and non-polar (Pro and Gly) properties, were co-incubated individually with 2OHE2, 4OHE2, or catechol. 2OHE2 and 4OHE2 are structural isomers with the same molecular weight and similar physical/chemical properties. On examination by LC-MS, conjugation with 4OHE2 could be detected as a mass shift of 284.1383 Da or 286.1554 Da on Lys, Cys, or His (Fig. 2). A mass shift of 284.1383 Da occurred mainly with Lys, and a shift of 286.1554 Da occurred mainly with Cys or His. However, both mass shifts could be found on all three AAs. CEs conjugation was not detected with the other AAs tested (data not shown), indicating that Lys, Cys, and His are the AAs residues that can be potentially modified. Same results were observed for 2OHE2 (Supplement Fig. S1), except that 2OHE2 was found to have a slightly lower reactivity with Lys and Cys and a slightly higher reactivity with His than 4OHE2.

As revealed by the fragmentation spectra acquired by CID on the modified AAs, covalent addition of the side chain of AAs to the C1 or C2 positions of the aromatic ring of 4OHE2 (Supplement Fig. S2a) yielded a mass shift of 286.1554 Da, or 284.1383 Da *via* further hydrogen abstraction from the saturated cyclic ring (Supplemental Fig. S2b). Since a mass shift of 284.1383 Da occurred mainly with Lys, we suspected that two closely eluted peaks noted in the extracted ion chromatograms (XICs) of the conjugated Lys (Fig. 2 left bottom) may arise from different isomeric products of hydrogen abstraction (different double bond location).

Pyridine dithioethylamine (PDA), which contains a disulfide bond, was reacted with 4OHE2 to test whether CEs can induce disulfide bond scission to expose thiols for modification. In the reaction mixture, a peak at a mass-to-charge ratio (m/z) of 398.1759, equivalent to the mass of 4OHE2 conjugated to the disulfide cleaved PDA, was detected (Fig. 2 right). This suggested that CEs oxidation may couple with reductive scission of the disulfide bond.

**Figure 3:**
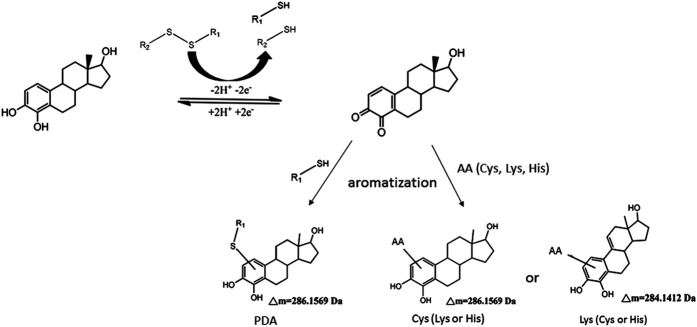
Proposed reaction scheme of CEs (4OHE2 as an example) with the side chain residue of AAs or with PDA via coupled disulfide reduction.

Based on these results, a reaction scheme is proposed in Fig. 3 for estrogenization of the side chains of Lys, Cys (free or disulfide-linked), and His, along with their corresponding mass shifts. As depicted, reductive scission of the disulfide bond contained in proteins or other molecules, such as PDA, may couple with the oxidation of CEs to CE-Qs, resulting in free thiol groups. Conjugation of CE-Qs with AAs or free thiols via Michael addition is followed by aromatization to regain the aromatic A-ring, resulting in PDA- or AA-conjugated CEs. In contrast to the CEs, catechol produced no conjugation products with any AAs (data not shown), indicating that catechol on its own is nonreactive without catalysis under ambient conditions. This is consistent with the cyclic voltammetry results (Fig. 1).

### Identification of estrogenized insulin

**Figure 4:**
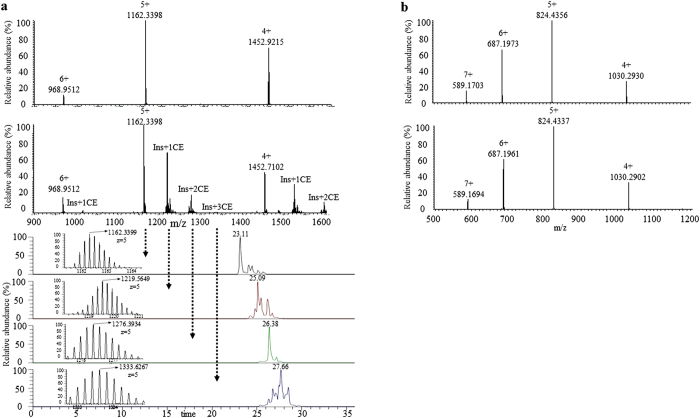
Protein estrogenization by co-incubation with 4OHE2 in PBS buffer. (**a**) MS spectra of the intact insulin (Humulin R) bearing +4, +5, and +6 charges before (top) and after (middle) co-incubation. Estrogenization was detected after co-incubation. The XICs (bottom) of insulin molecules (bearing the +5 charge state) adducted by different number (0–4) of 4OHE2 molecules. Retention times increased in proportion to the number of conjugated 4OHE2 molecules. Insets show the isotopic pattern of the precursor ions. (**b**) MS spectra of the intact teriparatide (Forteo) bearing +4, +5, +6, and +7 charges before (top) and after (bottom) co-incubation. Estrogenization was not detected after co-incubation.

To test whether insulin molecules can be estrogenized spontaneously, similarly to AAs, insulin (Humulin R) was reacted with 4OHE2 by co-incubation. The reaction mixtures were analyzed by nanoLC-MS^2^, both prior to enzyme digestion (top-down analysis) and immediately afterwards (bottom-up analysis). As shown in Fig. 4a (top), one or more 4OHE2 molecules were conjugated to intact insulin bearing different charge states (+4, +5, or +6 charges). The XICs of insulin bearing the +5 charges (bottom of Fig. 4a) indicated that retention times increased in proportion to the number of conjugated 4OHE2 molecules. As estimated from the ion intensity ratio of the modified *versus* unmodified insulin in the XICs (Supplement Fig. S3), co-incubation of an equal amount of insulin and 4OHE2 (100 μg each) resulted in a substantial percentage (>30%) of the insulin becoming estrogenized at 24 h. The percentage of estrogenized insulin increased with the amount of 4OHE2 in the solution (data not shown). After removal of free 4OHE2, the modified insulin was stable under storage at −4 °C for more than 8 days (Supplement Fig. S3).

We have also examined estrogenization of another recombinant protein drug, teriparatide (Forteo), by co-incubation with 4OHE2 under identical experimental conditions. Teriparatide comprises AA sequence 1–34 of human parathyroid hormone, and it is used to treat postmenopausal women with osteoporosis. No estrogenized form of teriparatide bearing different charge states (+4, +5, +6, or +7 charges) could be detected (Fig. 4b), indicating that teriparatide may not be susceptible to estrogenization.

**Figure 5:**
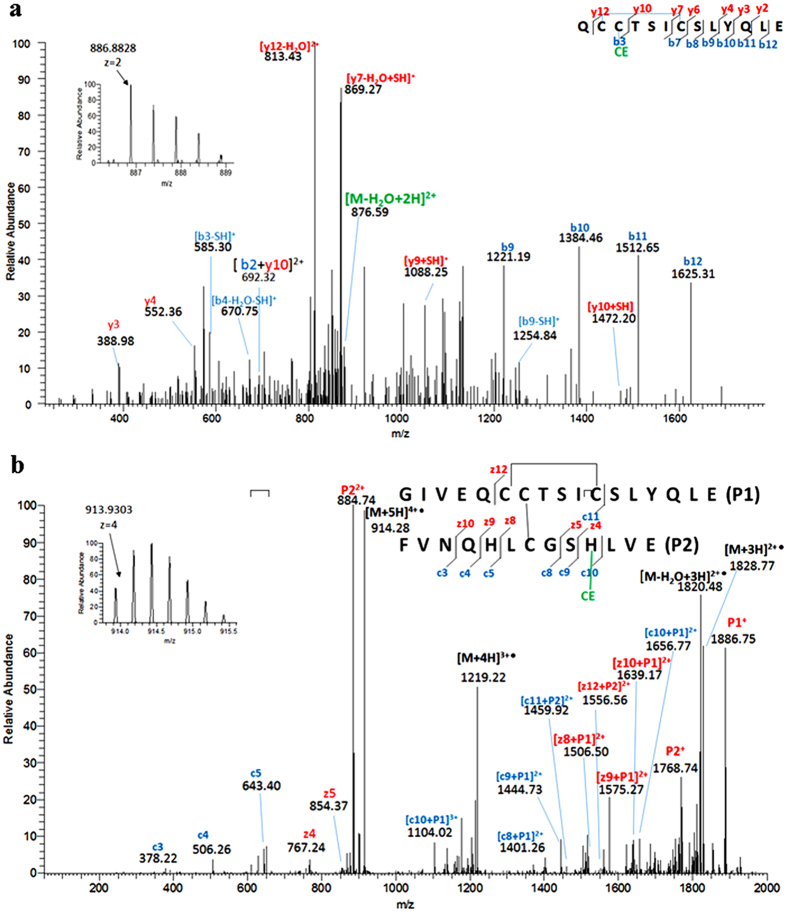
LC-MS2 Identification and verification of 4OHE2 modification sites on insulin. CID-MS spectra of the (**a**) AC7 and (**b**) BH10 -modified peptide. Insets show the isotopic pattern of the precursor ion.

Following digestion of Humulin R insulin with the endoproteinase GluC, the resulting mixture containing modified and unmodified Humulin R insulin was analyzed by data-dependent LC-MS-CID scans and targeted CID/ETD fragmentation. Four estrogenized peptides were identified, three of which were disulfide-linked (Table 1). These peptides covered the complete insulin sequence. Based on the b/y fragment ions acquired by CID shown in Fig. 5, estrogenization sites were unambiguously assigned to Cys7 in the A chain (A:Cys7) (Fig. 5a), Cys7 in the B chain (B:Cys7) (Supplemental Fig. S3a), and Lys29 in the B chain (B:Lys29) of insulin (Supplemental Fig. S3b). Identification of the modification site at His10 in the B chain (B:His10) from a disulfide-linked precursor ion was achieved by the detection of [c10 + P1]^2+^, [c9 + P1]^2+^, and z4 fragments cleaved by ETD (Fig. 5b). As noted above, ETD cleaved the inter-chain disulfide linkage in the gas phase to release P1 and P2, making sequence assignment and annotation unambiguous. Unmodified peptide counterparts were also detected, except the one spanning B:Cys7 (FVNQHL^CE^CGSHLVE), allowing further validation of our assignments based on the shifts of the precursor ion masses and retention times (Table 1).

### CD spectroscopy and molecular modeling on estrogenized insulin

**Figure 6:**
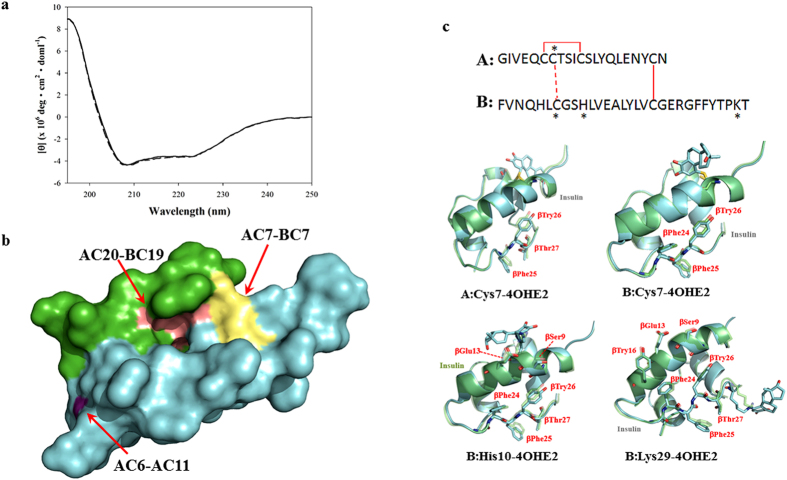
Structural changes in insulin upon estrogenization. (**a**) CD spectra of Ins (solid line) or CE-Ins (dashed line). Estrogenization has no effect on the secondary structure of insulin. (**b**) Exposed disulfide linkages, AC20–BC19 (pink), AC6–AC11 (purple), and AC7–BC7 (yellow), of insulin A chain (blue) and B chain (green). (**c**) Superimposed molecular models of non-estrogenized insulin (green) and estrogenized insulin (blue) with a single CE(4OHE2) modification site on either AC7, BC7, BH10, or BK29 residues (indicated as * in the sequence), respectively.

Estrogenization is likely to affect the secondary structure of proteins, or to block their binding domains. A mixture (CE-Ins) composed of un-modified and estrogenized insulin was prepared by co-incubation of the native Humulin R insulin with 4OHE2, as described above, yielding ~30% modified product. We compared the CD spectra of unmodified insulin (Ins) and CE-Ins. The absence of major differences in those spectra (Fig. 6a) indicated that estrogenization had no effect on the secondary structure of insulin. Molecular modeling was then used to study potential hindrance of receptor binding by the site-specific estrogenization of insulin identified here. Humulin R insulin is a monomer composed of A (21 AA) and B (30 AA) chains connected by two intermolecular disulfide linkages (A:Cys7–B:Cys7 and A:Cys20–B:Cys19). An intramolecular disulfide linkage (AC6–AC11) is also present. Based on molecular modeling (Fig. 6b), the surface exposure of the intermolecular disulfide linkage (A:Cys7–B:Cys7) is about 53.4 Å^2^, which is much greater than the 4.8 Å^2^ exposure of the intramolecular disulfide linkage (A:Cys6–A:Cys11) or the 19.4 Å^2^ exposure of the disulfide linkage (A:Cys20–B:Cys19). When models of insulin molecules modified by 4OHE2 at A:Cys7, B:Cys7, B:His10, or B:Lys29 were superimposed with native insulin (Fig. 6c), the orientation of some B chain AAs appeared to be altered. These included Ser9, Glu13, Tyr16, Phe24, Phe25, Try26, or Thr27, all of which contact the insulin receptor^19^.

### Functional assays on estrogenized insulin

**Figure 7:**
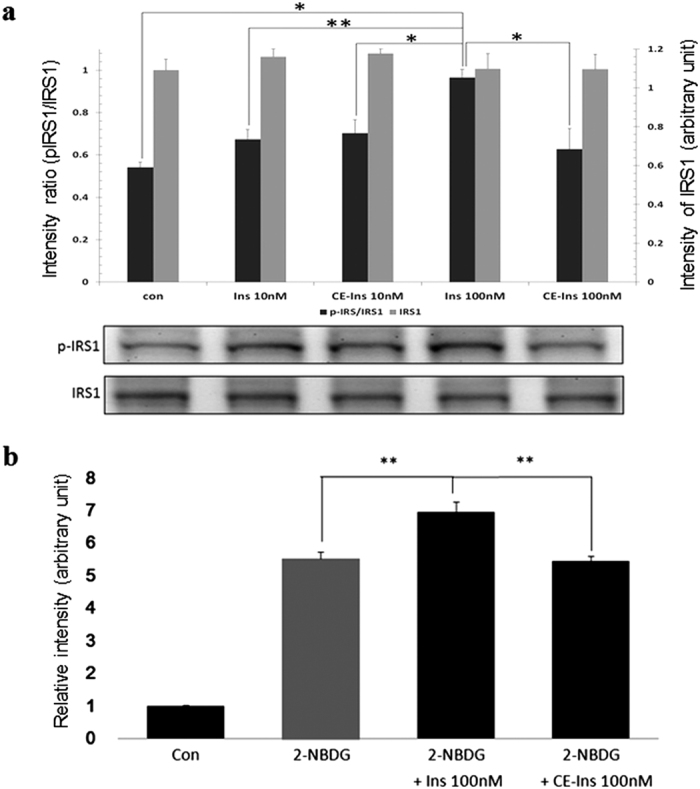
Functional assays of insulin estrogenization. (**a**) Insulin estrogenization reduces the efficacy of insulin signaling. MCF-7 cells were treated with the PBS buffer as the control (con), 10 or 100 nM of Ins or CE-Ins solution. Western blot of IRS phosphorylation was detected using primary antibodies against pIRS1 (pSer636/pSer639) and IRS1 protein, respectively. Results are expressed as the mean with standard deviation (SE) derived from four separate experiments (n = 4). (**b**) Insulin estrogenization reduces the efficacy of insulin-induced glucose uptake. MCF-7 cells were incubated with the PBS buffer as the control (Con), 2-NBDG (20 mM), 2-NBDG (20 mM) and Ins (100, 1000 nM), or 2-NBDG (20 mM) and CE-Ins (total insulin concentration of 10 or 100 nM) for 30 min. The level of glucose in the cell was detected as the fluorescence intensity. Results of eight experiments (n = 8) are expressed as the mean and standard deviation (SE). Significant differences were indicated as *(p < 0.05) or **(p < 0.01).

MCF-7 cells were challenged with native and modified insulin, and the phosphorylation of insulin receptor substrate-1 (IRS-1) at Ser636 or Ser639 was examined to test whether estrogenization of insulin altered its biological activity. As shown in Fig. 7a, when cells were treated with 100 nM of Ins, a 20% increase in IRS-1 phosphorylation was detected by western blotting. This effect was not observed in cells treated with 100 nM CE-Ins (Fig. 7a), even though the levels of IRS-1 in cells treated with either Ins or CE-Ins were similar to those in the control (Fig. 7a). As shown in Fig. 7b, although a 20% increase (*vs.* the control) in glucose uptake was detected in MCF-7 cells treated with 100 nM Ins, no change occurred in samples treated with 100 nM CE-Ins, indicating that the estrogenization of insulin prevented insulin-dependent glucose uptake.

## Discussion

Although catechol, a redox moiety contained in CEs, must be activated by enzymes or reactive oxygen species before binding, we found that CEs conjugate to AAs or proteins at 37 °C without catalysis. Moreover, CEs are capable of causing disulfide bond-scission, exposing free thiols for modification, possibly through CE oxidation-coupled disulfide cleavage followed by Michael addition (Fig. 3). Cyclic voltammetry measurements (Fig. 1) demonstrate that the oxidation potential of 4OHE2 is 50% lower than that of catechol, indicating that the oxidation of 4OHE2 is thermodynamically easier to achieve than that of catechol. Moreover, the large decrease in peak-to-peak separation indicates that the overall process is kinetically much faster for 4OHE2 than catechol. Thus, while the conjugation of CEs occurs under ambient conditions without catalysis, the conjugation of catechol does not. This may be due to an electron-withdrawing effect by the substituted cyclic-alkyl chains on the catechol ring of CEs, decreasing the pKa values of the phenolic hydroxyl groups, shifting the reaction to the right (Fig. 3), and favoring the formation of CE-Q. Because the catechol moiety is generally believed to be the reactive site on CEs, it is plausible that CEs are more reactive than expected.

Our data show that cysteine, lysine, and histidine are the target residues for estrogenization, which is consistent with observations on human sera^8^. Insulin molecules contain 4 CE modification sites, which is quite substantial for a small protein. In addition, compared to many post-translational modifications such as phosphorylation or glycosylation, estrogenization of >30% of the insulin molecules under ambient conditions appears to be quite high. In contrast, we observed no estrogenization of another protein, teriparatide (Forteo), upon co-incubation with 4OHE2 (Fig. 4b).

Estrogenization sites are highly dependent on the accessibility and reactivity of target residues on a protein. For example, C34, the only free cysteine residue and a frequent conjugation site of human serum albumin^19^, was identified as a major estrogenization target site^8^. Insulin contains three disulfide linkages, and the estrogenized disulfide linkage (A:Cys7–B:Cys7) has a much greater exposed surface area (53.4 Å^2^) than the other two unmodified disulfide linkages (A:Cys6–A:Cys11 (4.8 Å^2^) and A:Cys20–B:Cys19 (19.4 Å^2^)).

The folding and/or higher-order structure of many globular proteins is determined by disulfide linkages. However, according to our CD data and modeling, estrogenization does not alter the secondary structure of insulin. The disulfide linkages that connect the insulin A and B chains, which are distinct peptides, have little effect on the conformation of those chains. Nevertheless, estrogenization of insulin on any of the four identified sites may alter the structure of its receptor-binding pocket. The insulin A chain has an amino-terminal helix (A1–A8) linked to an antiparallel carboxy-terminal helix (A12–A20). The insulin B chain has a central helix (B8–B19) flanked by extended amino- and carboxy-terminal strands. Insulin interacts with the primary binding site on its receptor (site 1; dissociation constant, 6.4 nM) composed of the carboxy-terminal α-chain and the central β-sheet of the first leucine-rich-repeat domain (L1–β2) of the other α-chain within the insulin receptor dimer^20^. The C-terminal end of the L1–β2 strand of the receptor engages with the insulin B-helix (B7–B21) but has no interaction with the insulin A chain. Moreover, the αCT segment displaces the C-terminal β-strand of the B chain (B26–B30) away from the hormone core upon receptor engagement. Insulin estrogenization at A:Cys7, B:Cys7, B:His10, or B:Lys29 (Fig. 6) alters the orientation of some B chain AAs that contact the insulin receptor, such as B:Ser9, B:Glu13, B:Tyr16, B:Phe24, B:Phe25, B:Try26, or B:Thr27^21^. Thus, the functional impact of estrogenization on insulin molecules should not be overlooked.

Endogenous insulin is produced by the pancreas and stored in the body as a Zn^2+^-stabilized hexamer. This hexamer is an inactive form possessing long-term stability. It remains to be investigated whether estrogenization affects insulin stability by disrupting the Zn^2+^-stabilized hexamer. However, biologically active insulin circulates as a Zn^2+^-free monomer that binds to insulin receptors, inducing numerous cascades, including glucose influx *via* translocation of the GLUT-4 transporter to the plasma membrane^21^,^22^. We demonstrated that estrogenization of either endogenous monomeric insulin or Humulin R insulin may reduce or block its action by affecting insulin signaling and insulin-dependent glucose uptake. These results imply that the estrogenization of insulin may prevent insulin from playing a role in IRS-1 phosphorylation. Thus, it is possible that insulin estrogenization may contribute to insulin resistance by disrupting insulin structures or by blocking insulin signaling. Insulin resistance has been partly attributed to be a cellular antioxidant defense^23^, and this coincides with the quenching mechanism for the reactive CE electrophiles that can bring about insulin estrogenization.

We established that CE is more reactive than would be expected based on the catechol moiety, and the molecular structure of insulin makes it highly susceptible to extensive estrogenization, leading to altered insulin action. Given the importance of estrogen metabolism and insulin signaling, as well as the putative structural changes associated with CE modifications, estrogenization may exert important effects on physiological or pharmacological functioning of insulin and is worth exploring further. We believe that the new data reported here will be valuable for conducting future studies.

## Methods

### Materials

Iodoacetamide, tris(2-carboxyethyl)phosphine, formic acid (FA), 2-hydroxyestradiol (2OHE2), 4-hydroxyestradiol (4OHE2), L-lysine, L-histidine, L-cysteine, L-arginine, L-tyrosine, L-serine, L-proline, L-glutamine,and L-glutamic acid were purchased from Sigma-Aldrich (St. Louis, MO, USA). Therapeutic insulin (Humulin R) and teriparatide (Forteo) were from Eli Lilly and Company (Indianapolis, IN, USA). Trichloroacetic acid, L-glycine, sodium dodecyl sulfate (SDS), sodium chloride, ammonium bicarbonate, and acetonitrile were purchased from JT Baker (Center Valley, PA, USA). Pyridine dithioethylamine (PDA) was obtained from Nanocs (New York, NY, USA). Glu-C was purchased from Roche Life Sciences (Indianapolis, IN, USA). Trypsin was purchased from Promega BioSciences (San Luis Obispo, CA, USA). 2-deoxy-2-[(7-nitro-2,1,3-benzoxadiazol-4-yl)amino]-D-glucose (2-NBDG) was from Life Technologies (Grand Island, NY, USA). The antibody against endogenous IRS1 phosphorylated at Ser636 or Ser639 (anti-pIRS1) was purchased from Abcam (Cambridge, MA, USA).

### Conjugation reactions

To conjugate AAs with CEs, 15 μL of a 10 mg/mL AA solution (containing either histidine, cysteine, lysine, arginine, glutamine, glutamic acid, tryptophan, proline, or glycine) in phosphate buffer (10 mM, pH = 7.4), was mixed with 10 μL of either 4OHE2 or 2OHE2 (5 mg/mL in acetonitrile), and diluted to 75 μL with the phosphate buffer. Acetonitrile was evaporated from the solution by purging with nitrogen gas until a final volume of ~52 μL was reached. The mixture was incubated at 37 °C for 50 min and then diluted with formic acid (0.1%) to a final volume of 1 mL.

Similar procedures were followed for the insulin conjugation experiments. Briefly, 20 μL of a 4OHE2 solution (5 μg/μL) were added to 100 μL of insulin (Humulin R) or teriparatide (Forteo) solution (1 μg/μL), and the mixtures were incubated at 37 °C for 24 h (or other specified times). Unbound 4OHE2 was removed by centrifugation (16,286 × *g*) through a 3 kDa cutoff spin column (Amicon Ultra 3 K, Merck Millipore, Darmstadt, Germany). The resulting mixture of insulin conjugation reaction (CE-Ins) composed of un-modified insulin and 4OHE2-conjugated insulin (>30%).

### Protein digestion

The adducted insulin solution prepared as described above was re-dissolved in 50 μL ammonium bicarbonate (50 mM, pH 8.3) and followed by GluC digestion at protein-enzyme ratios of 1:25 or 1:50. The digestion reaction proceeded for 18 h at room temperature and the digested product was dried under a vacuum.

### Cyclic voltammetry

For cyclic voltammetry measurements, 4OHE2 (287.5 μg/mL) and catechol (55 μg/mL) solutions were prepared, dissolved in de-ionized water containing 0.1 M sodium chloride. Measurements were performed at an electrochemical work station (CHI405, CHI Instruments, Inc. Austin, TX, USA) using a glassy-carbon working electrode, a Pt wire counter-electrode, and a Ag/AgCl reference electrode with a scan speed of 5 mV/min, a scan range from −0.2 to 0.8 V, a sampling interval of 0.001 V, and scan sensitivity (A/V) of 1 × 10^−6^.

### LC-MS/MS Analysis

For AA analysis, 10 μL of the prepared AA sample was injected onto an HPLC column (1.0 mm i.d. × 10 cm, 1.7 μm C18, BEH130, Waters Corporation, Milford, MA, USA) coupled online to a Q-TOF instrument (ACQUITY UPLC and Xevo G2 QTof, Waters Corporation, Milford, MA, USA), and eluted over 40 min using the following gradient: 0.1% FA/acetonitrile in 0.1% FA gradient at a flow rate of 70 μL/min: 0–5 min, 5% acetonitrile in 0.1% FA; 5–10 min, 5 to 30% acetonitrile in 0.1% FA; 10–25 min, 30 to 40% acetonitrile in 0.1% FA; 25–27 min, 40 to 90% acetonitrile in 0.1% FA; 27–30 min, 90% acetonitrile in 0.1% FA; 30–32 min, 90 to 5% acetonitrile in 0.1% FA; and 32–40 min, 5% acetonitrile in 0.1% FA.

### NanoLC-MS/MS Analyses

A solution containing 0.01 μg of intact insulin or teriparatide was injected onto a nano-HPLC system (ACQUITY UPLC) equipped with a pre-column (180 μm i.d. × 2 cm, 5 μm C18, Waters Corporation) and a nano column (75 μm i.d. × 10 cm, 1.7 μm C18, BEH130 Waters Corporation) coupled online to a LTQ-Orbitrap XL mass spectrometer (Thermo Fisher Scientific, San Jose, CA, USA). The sample was eluted initially over 5 min for sample loading with a mobile phase of 5% acetonitrile in 0.1% FA at a flow rate of 5 μL/min. This was followed by a 40 min 0.1% FA/acetonitrile in 0.1% FA gradient at a flow rate of 300 nL/min: 0–5 min, 5% acetonitrile in 0.1% FA; 5–15 min, 5 to 30% acetonitrile in 0.1% FA; 15–25 min, 30 to 50% acetonitrile in 0.1% FA; 25–27 min, 50 to 95% acetonitrile in 0.1% FA; 27–29 min, 95% acetonitrile in 0.1% FA; 29–32 min, 95 to 5% acetonitrile in 0.1% FA; and 32–40 min, 5% acetonitrile in 0.1% FA.

For peptide analyses, 0.5 μL of the re-dissolved digest solution was injected. The elution profile and MS data acquisition method were described previously^8^. Briefly, the sample was eluted initially over 5 min for sample loading using 5% acetonitrile in 0.1% FA at a flow rate of 5 μL/min and followed by a 70 min 0.1% FA/acetonitrile in 0.1% FA gradient at a flow rate of 300 nL/min. MS data were acquired using the data-dependent mode where one full scan with m/z 300–2000 in the Orbitrap (R = 60,000 at m/z 400) at a scan rate of 30 ms/scan was followed by the five most intense peaks for fragmentation with a normalized collision energy value of 35% in the LTQ. A repeat duration of 30 s was applied to exclude the same m/z ions from being re-selected for fragmentation.

### CD Spectroscopy

Far-UV CD spectra were recorded at 25 °C on a JASCO J-815 spectropolarimeter equipped with a temperature-controlling liquid system. Samples of Ins or CE-Ins (prepared by conjugation reactions described above) were diluted in 2 mM PBS to a final concentration of 4.0 μM. Spectral analysis occurred over the wavelength range of 190–260 nm, in cuvettes with a 2-mm path length, at 0.2-nm intervals with a 4 s integration time and a bandwidth of 2.0 nm. Photomultiplier absorbance did not exceed 600 V in the analyzed spectral region. All of the measurements were performed under nitrogen flow. Each scan was repeated ten times and an average was obtained. The results were expressed as mean residue ellipticities [θ] in units of degrees cm^2^ dmol^−1^, which is defined as [θ] = 10^6^_obs_ (*lc*)^−1^, where θ_obs_ is the observed ellipticity in degrees, *c* is the concentration in moles per liter, and *l* is the length of the light path in centimeters.

### Cell culture

Human breast cancer cells (MCF-7) were obtained from the Bioresource Collection and Research Center (BCRC, Hsinchu, Taiwan). Cells were grown in 10 cm dishes with phenol red-free Dulbecco’s modified Eagle’s medium (DMEM) in sodium bicarbonate buffer (17.8 mM, pH 7.2) containing 1% (v/v) streptomycin/penicillin/amphotericin B (Antibiotic-Antimycotic, Life Technologies) and 10% fetal bovine serum. Cells were incubated in a humidified 5% CO_2_ incubator at 37 °C until confluence. The cells were then starved for 24 h, followed by treatment with Ins or CE-Ins solution to a total insulin concentration of 10 or 100 nM at 37 °C for 24 h.

### Cell signaling

MCF-7 cells (2 × 10^6^), treated with Ins or CE-Ins solution, were collected in 350 μL of RIPA buffer (150 mM NaCl, 1% NP-40, and 50 mM Tris-HCl, pH 7.4) containing one protease-inhibitor cocktail tablet (Complete, Sigma-Aldrich). The cells were lysed by gentle rotation for 30 min at 4 °C, followed by passing the sample through a 25-gauge needle to dissociate the bound DNA. For western blotting, 30 μL of the whole cell lysate, containing 100 μg of total protein, was separated by 8% SDS-PAGE at 100 V for ~4 h, and then transferred overnight from the gel to a 0.22 μm PVDF membrane (Stratagene, La Jolla, CA, USA). The membrane was blocked for 1 h with 5% BSA in TBST buffer (150 mM NaCl, 0.05% Tween 20, and 50 mM Tris, pH 7.4), and then incubated overnight with a primary antibody against Ser636 or Ser639 of IRS-1, or IRS-1 protein (Abcam). The secondary antibody (anti-rabbit) was co-incubated with the sample for 1 h. The blot was developed using an enhanced chemiluminescence detection reagent (Amersham ECL Plus; GE Healthcare, Piscataway, NJ, USA), and the spot intensity was digitized using a computerized image analyzer.

### Glucose uptake measurements

MCF-7 cells (2 × 10^6^), treated with Ins or CE-Ins solution, were cultured for 4 h without glucose. Cells were then trypsinized, collected in a photo-protected (brown) microcentrifuge tube, and washed with PBS. This was followed by centrifugation (57.2 × *g*) for 5 min and three washes with ice-cold PBS. For glucose uptake experiments, 2-NBDG was dissolved in water, added to the cells at a final concentration of 20 mM. Cells were incubated with 2-NBDG for 30 min at 37 °C. Uptake was terminated by the rapid removal of the assay buffer, followed by three washes with ice-cold PBS. Cells were centrifuged briefly and then used for fluorescence measurements. Unstimulated cells treated with the same amount of water used to dissolve 2-NBDG, were used to determine the background fluorescence. A second control consisted of 2-NBDG added to unstimulated cells. In case of the presence of trace amount of 4OHE2 in CE-Ins solution, cells treated with 4OHE2 (35 μM) were investigated, confirming that no change in glucose uptake by 4OHE2 occurs compared to the 2-NBDG background control.

### Molecular modeling

Molecular models of insulin were constructed using the SWISS-MODEL protein modeling server, based on an X-ray crystal structure of human insulin (PDB code: 3W11). Models of insulin estrogenized with 4OHE2 at specific sites were individually built. Energy minimization was carried out in Discovery Studio 2.5 software (Accelrys, Burlington, MA, USA) using the CHARMM force field, and in LibDock. Molecular models of estrogenized insulin were superimposed individually on the molecular model of native insulin, and displayed using PyMol software (http://www.pymol.org/).

### Statistics

All data were analyzed using Excel (Microsoft Excel, Redmond, WA, USA) and are presented as means ± SE. Results were compared by one-way analysis of variance (ANOVA). Differences were considered significant at P < 0.05 or less.

## Additional Information

**How to cite this article**: Ku, M.-C. *et al*. Site-specific covalent modifications of human insulin by catechol estrogens: Reactivity and induced structural and functional changes. *Sci. Rep.* **6**, 28804; doi: 10.1038/srep28804 (2016).

## Supplementary Material

Supplementary Information

## Figures and Tables

**Table 1:**
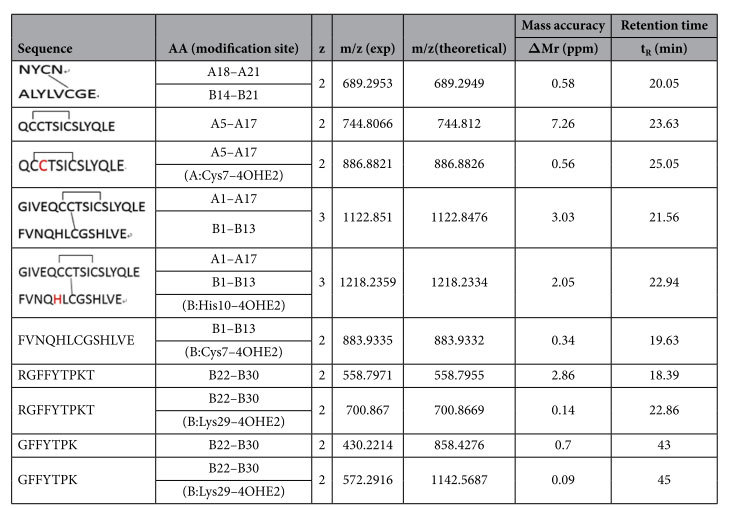
Peptides of insulin identified by LC-MS and 4OHE2 modification sites.
