# Supplementary material for: Site-specific covalent modifications of human insulin by catechol estrogens: Reactivity and induced structural and functional changes
Source: Sci Rep. 2016 Jun 29;6:28804. doi: 10.1038/srep28804 (PMC4926285; doi:10.1038/srep28804)

Site-specific covalent modifications of human insulin by catechol estrogens: Reactivity and induced structural and functional changes

Ming-Chun Ku,[1#] Chieh-Ming Fang,[1#] Juei-Tang Cheng,[2] Huei-Chen Liang,[1] Tzu-Fan Wang,[1] Chih-Hsing Wu,[3] Chiao-Chen Chen,[1] Jung-Hsiang Tai,[4] and Shu-Hui Chen[1*]

[1]Department of Chemistry, National Cheng Kung University, Tainan, Taiwan, ROC

[2]Department of Medical Research, Chi-Mei Medical Center, Yong-Kang, Tainan, Taiwan, ROC

[3]Department of Family Medicine, College of Medicine, National Cheng Kung University Hospital, Tainan, Taiwan, ROC

[4]Division of Infectious Diseases and Immunology, Institute of Biomedical Sciences, Academia Sinica, Taipei, Taiwan

[#]These authors contributed equally

[*]Corresponding author: shchen@mail.ncku.edu.tw

Supplement Fig. S1 Covalent conjugation with 2OHE2 via co-incubation in PBS buffer. a. XICs of 2OHE2-conjugated lysine (left), cysteine (middle) and histidine (right) before co-incubation (top) and after co-incubation (bottom). Modified AAs were all detected after co-incubation. Multiple peaks having an identical mass were noted in that of 2OHE2-conjugated Lys (left), possibly arising from different steric isomers of the product.

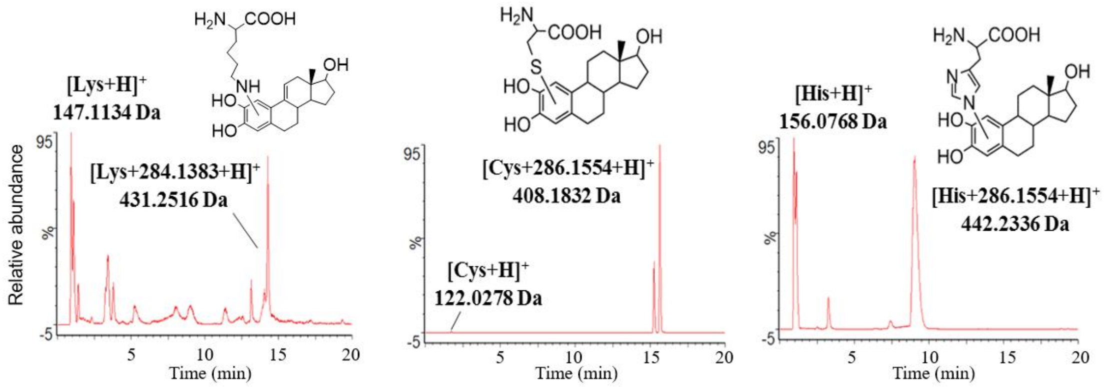

Supplement Fig. S2 CID-MS spectra of the (a) 4OHE2-modified lysine and (b) 4OHE2-modified cysteine. Insets show the isotopic pattern of the precursor ion.

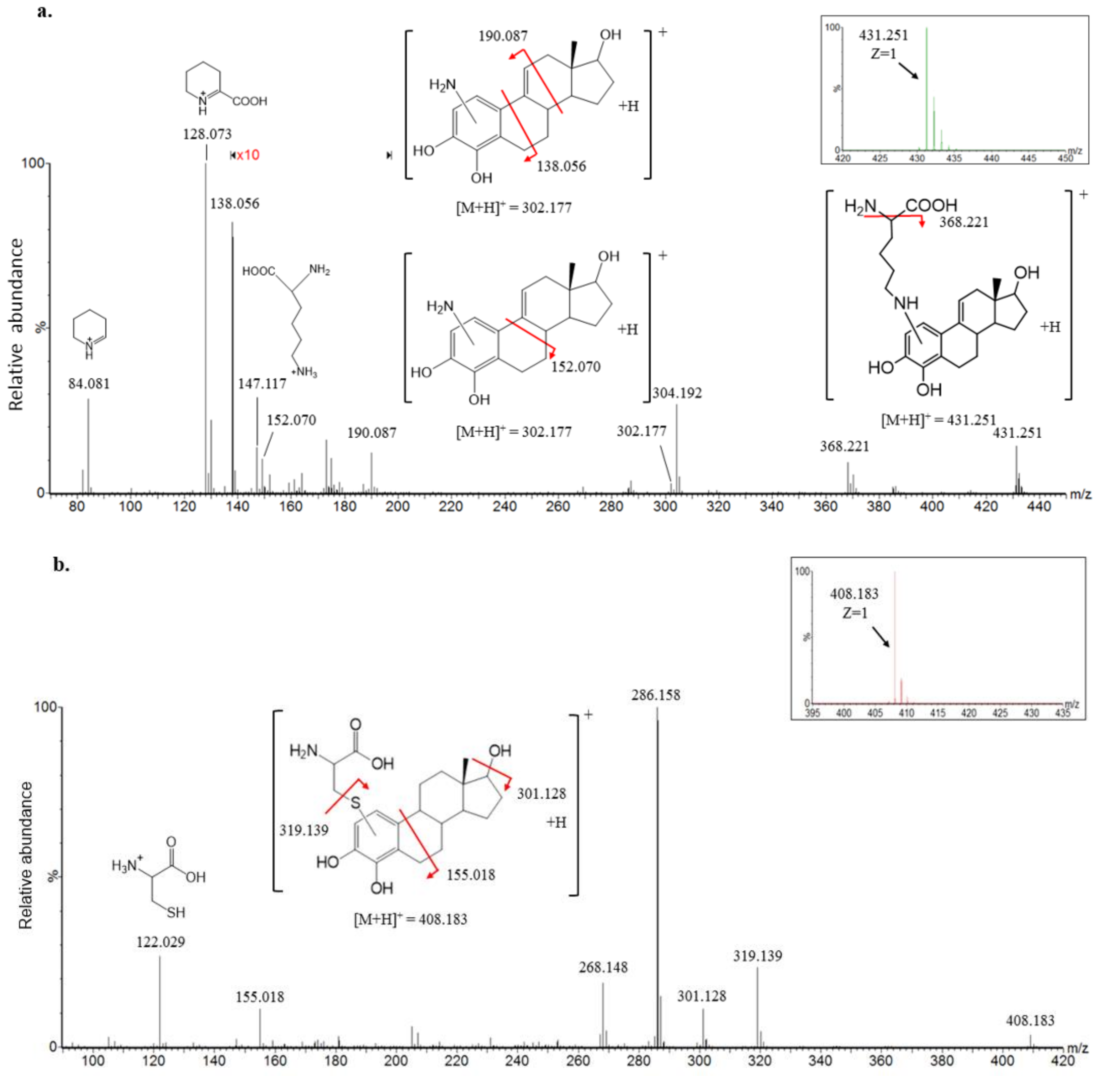

Supplement Fig. S3. Stability study for the estrogenized insulin. Plot of the ion intensity ratio of 4OHE2-adducted insulin (sum of 1-3 4OHE2) divided by insulin versus the time under 37°C co-incubation (before the dashed line) or -4°C storage (after the dashed line). The unbound free 4OHE2 was removed by a 3-kDa molecular cutoff centrifuge device before the storage. An equal amount (100 μg) of insulin and 4OHE2 was used for co-incubation.

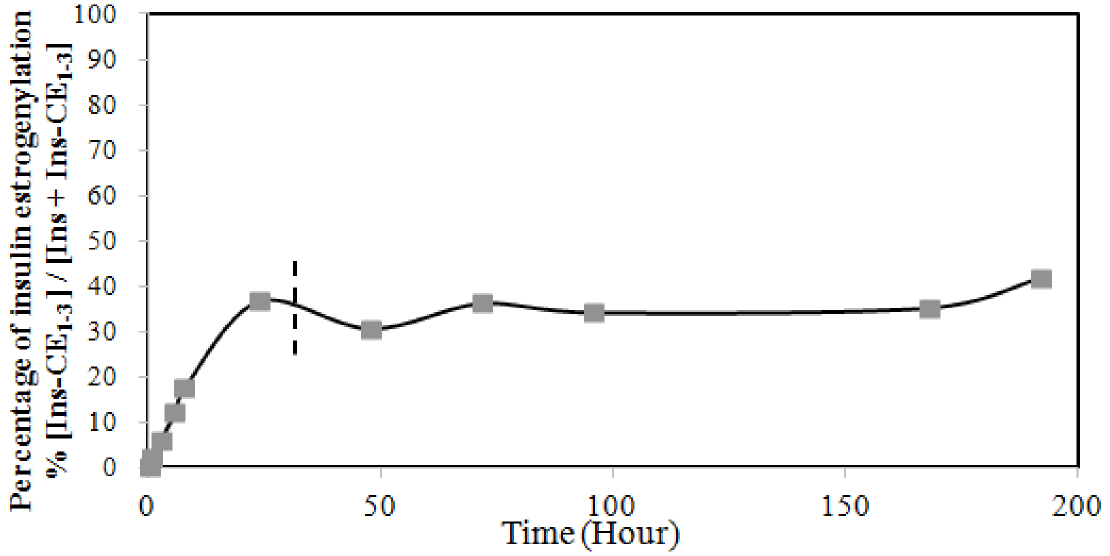

Supplement Fig. S4 LC-MS2 Identification and verification of 4OHE2 modification sites on insulin. CID-MS spectra of the (a) BC7 and (b) BK29 -modified peptide. Insets show the isotopic pattern of the precursor ion.

a

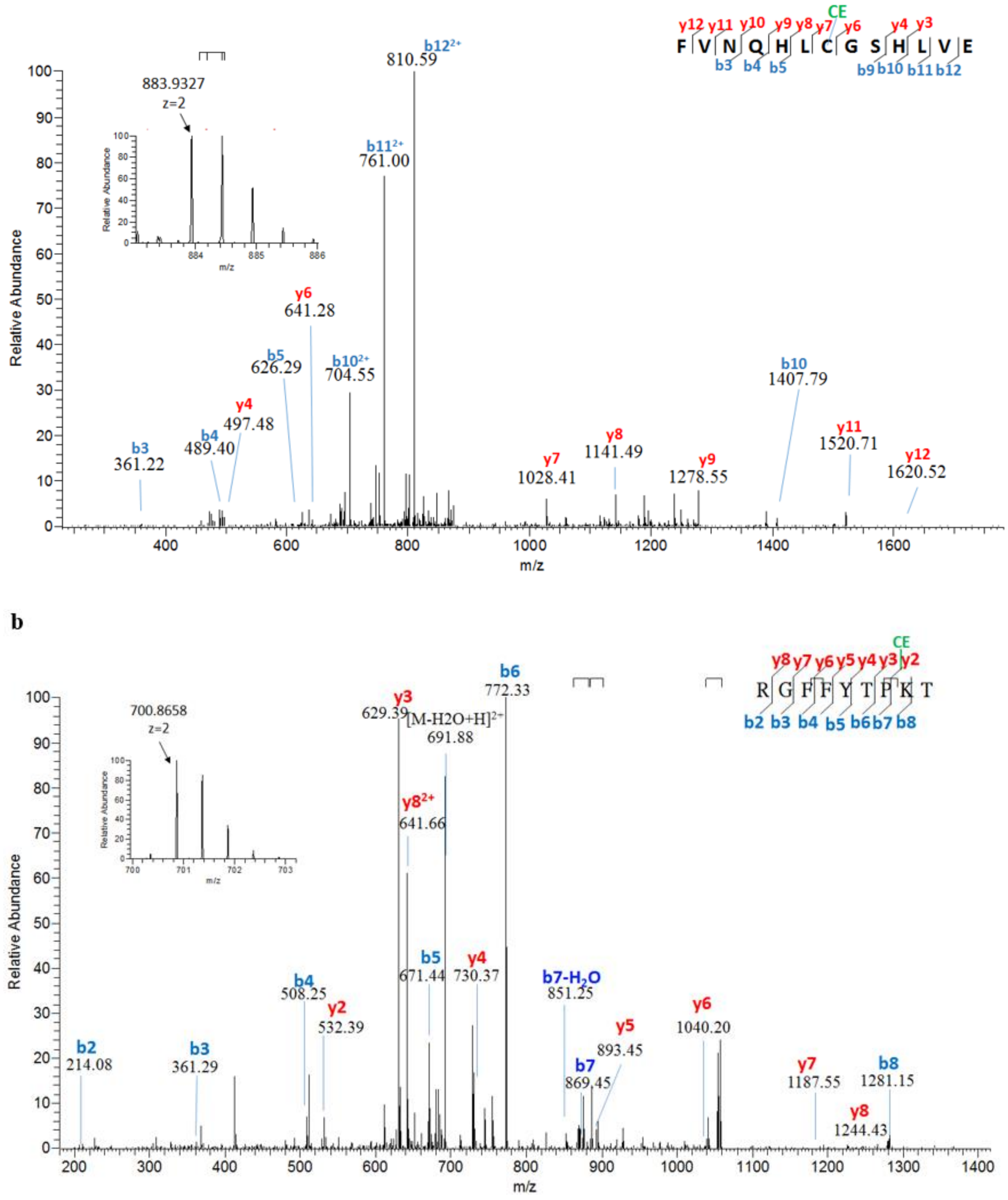

Supplement: Supplementary Information [file srep28804-s1.pdf]
